# The P-type ATPase inhibiting potential of polyoxotungstates†
†Electronic supplementary information (ESI) available: Positive control experiment for Na^+^/K^+^-ATPase inhibition of ouabain. See DOI: 10.1039/c7mt00279c


**DOI:** 10.1039/c7mt00279c

**Published:** 2018-01-05

**Authors:** Nadiia Gumerova, Lukáš Krivosudský, Gil Fraqueza, Joscha Breibeck, Emir Al-Sayed, Elias Tanuhadi, Aleksandar Bijelic, Juan Fuentes, Manuel Aureliano, Annette Rompel

**Affiliations:** a Universität Wien , Fakultät für Chemie , Institut für Biophysikalische Chemie , Althanstraße. 14 , 1090 Wien , Austria . Email: annette.rompel@univie.ac.at ; www.bpc.univie.ac.at; b Centre of Marine Sciences , University of Algarve , 8005-139 Faro , Portugal; c Institute of Engineering , University of Algarve , 8005-139 Faro , Portugal; d Faculty of Sciences and Technology , University of Algarve , 8005-139 Faro , Portugal . Email: maalves@ualg.pt

## Abstract

Polyoxometalates (POMs) are transition metal complexes that exhibit a broad diversity of structures and properties rendering them promising for biological purposes. POMs are able to inhibit a series of biologically important enzymes, including phosphatases, and thus are able to affect many biochemical processes. In the present study, we analyzed and compared the inhibitory effects of nine different polyoxotungstates (POTs) on two P-type ATPases, Ca^2+^-ATPase from skeletal muscle and Na^+^/K^+^-ATPase from basal membrane of skin epithelia. For Ca^2+^-ATPase inhibition, an *in vitro* study was performed and the strongest inhibitors were determined to be the large heteropolytungstate K_9_(C_2_H_8_N)_5_[H_10_Se_2_W_29_O_103_] (Se_2_W_29_) and the Dawson-type POT K_6_[α-P_2_W_18_O_62_] (P_2_W_18_) exhibiting IC_50_ values of 0.3 and 0.6 μM, respectively. Promising results were also shown for the Keggin-based POTs K_6_H_2_[CoW_11_TiO_40_] (CoW_11_Ti, IC_50_ = 4 μM) and Na_10_[α-SiW_9_O_34_] (SiW_9_, IC_50_ = 16 μM), K_14_[As_2_W_19_O_67_(H_2_O)] (As_2_W_19_, IC_50_ = 28 μM) and the lacunary Dawson K_12_[α-H_2_P_2_W_12_O_48_] (P_2_W_12_, IC_50_ = 11 μM), whereas low inhibitory potencies were observed for the isopolytungstate Na_12_[H_4_W_22_O_74_] (W_22_, IC_50_ = 68 μM) and the Anderson-type Na_6_[TeW_6_O_24_] (TeW_6_, IC_50_ = 200 μM). Regarding the inhibition of Na^+^/K^+^-ATPase activity, for the first time an *ex vivo* study was conducted using the opercular epithelium of killifish in order to investigate the effects of POTs on the epithelial chloride secretion. Interestingly, 1 μM of the most potent Ca^2+^-ATPase inhibitor, Se_2_W_29_, showed only a minor inhibitory effect (14% inhibition) on Na^+^/K^+^-ATPase activity, whereas almost total inhibition (99% inhibition) was achieved using P_2_W_18_. The remaining POTs exhibited similar inhibition rates on both ATPases. These results reveal the high potential of some POTs to act as P-type ATPase inhibitors, with Se_2_W_29_ showing high selectivity towards Ca^2+^-ATPase.

## Abstract 2

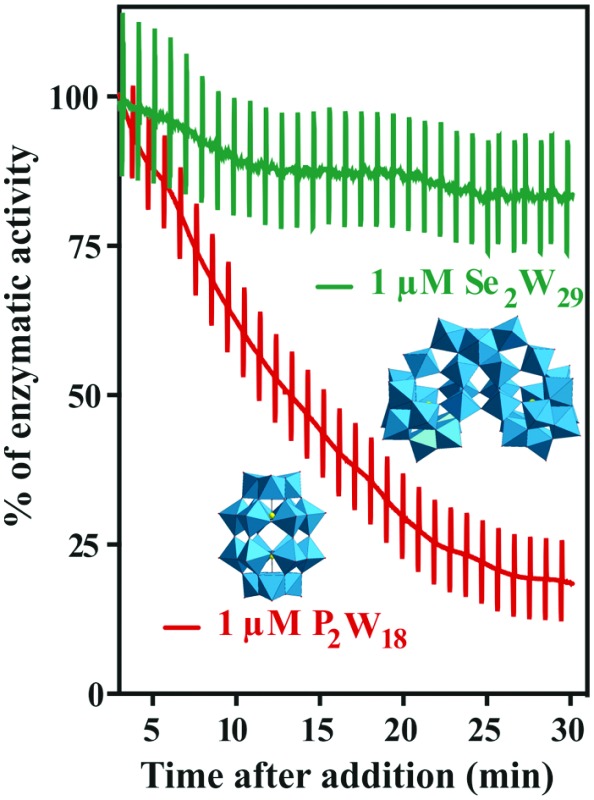
Polyoxometalates (POMs) are transition metal complexes that exhibit a broad diversity of structures and properties rendering them promising for biological purposes.

##

Significance to metallomics

We studied the inhibitory effects of nine different polyoxotungstates (POTs) on P-type ATPases *in vitro* (Ca^+^-ATPase) and *ex vivo* (Na^+^/K^+^-ATPase). The study reveals that some POTs like the Dawson anion [P_2_W_18_O_62_]^6–^, which was highly active *in vitro* and *ex vivo*, are potent ATPase inhibitors. Furthermore, there is a charge density-activity correlation for the most potent POTs (IC_50_ < 16 μM), namely Se_2_W_29_, P_2_W_18_, CoW_11_Ti, SiW_9_ and P_2_W_12_. As P-type ATPases represent pharmacologically important targets due to their important role in health and disease, the here reported bioactive POTs should be considered as possible future metallodrugs.

## Introduction

**Fig. 1:**
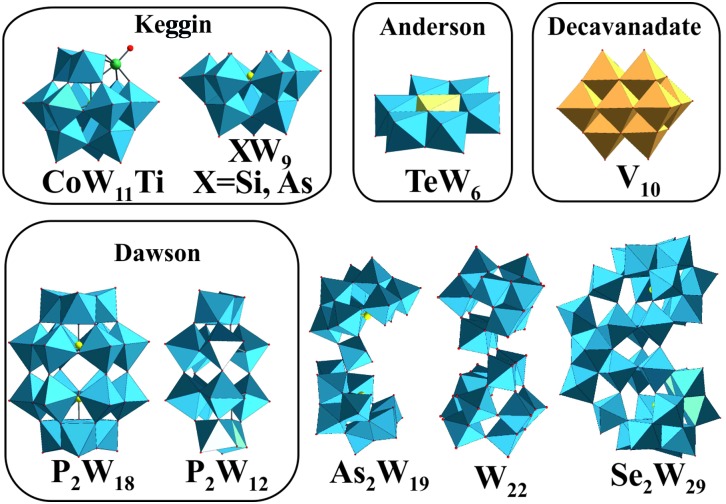
Structures of the investigated POTs (Table 1) and [V_10_O_28_]^6–^. Color code: WO_6_, blue polyhedra; VO_6_, dark yellow polyhedra; heteroatom, light yellow sphere or polyhedra; Ti as substituted atom, green sphere.

Polyoxometalates (POMs) are metal clusters^1^ that exhibit a broad diversity of structures and outstanding properties leading to their application in various fields such as catalysis,^2^,^3^ photochemistry,^4^ material science,^5^,^6^ macromolecular crystallography^7^–^15^ and medicine.^16^–^22^ POMs can be divided into isopolyanions (IPAs), which consist only of one type of metal atom (M = addenda atom), [M_*m*_O_*y*_]^*q*–^, and heteropolyanions (HPAs), which contain one or more additional elements (X = heteroatom), [X_*r*_M_*m*_O_*y*_]^*q*–^. The most common representative of IPAs is the Lindqvist structure, whereas the well-known Dawson, Keggin and Anderson archetypes belong to the HPAs (Fig. 1). POM research represents an emerging field and especially bioactive POMs are getting more and more attractive due to their ability to interact with important enzymes like alkaline phosphatases, ecto-nucleotidases and ATPases and their potential to interfere with specific cellular processes, such as mitochondria respiration.^21^–^24^ POMs like decavanadate or Keggin-type polyoxotungstates (POTs) and polyoxomolybdates are currently the focus of biological and biomedical research as they show promising antibacterial and antidiabetic activities,^21^,^22^,^24^–^28^ whereas only few biological studies exist for other POM archetypes such as the Anderson structure.^29^

The main role of the sarcoplasmic reticulum (SR) Ca^2+^-ATPase is translocation of cellular Ca^2+^ from the cytoplasm to the SR, which is involved in muscle relaxation.^30^,^31^ However, Ca^2+^-ATPase is globally associated with cellular calcium homeostasis, a process of ion transport that is coupled with ATP hydrolysis. ATP hydrolysis follows a well-known mechanism traversing at least four intermediate steps and two protein conformations, namely E1 and E2, with E1 being the conformation with high affinity for the exported substrate and E2 the form with high affinity for the imported substrate.^30^,^31^ As SR vesicles from skeletal muscle contain a large amount of Ca^2+^-ATPase, they represent a useful *in vitro* model to study the effects of drugs and POMs on calcium homeostasis.^32^,^33^ To our knowledge, only a few POMs, such as decavanadate (V_10_) and decaniobate (Nb_10_), were described to be potent non-competitive inhibitors (IC_50_ = 15 and 35 μM, respectively) of the hydrolytic activity of SR Ca^2+^-ATPase.^33^ Na^+^/K^+^-ATPase transports Na^+^ out of the cell while pumping K^+^ into cells and is thus responsible for the ionic and osmotic balance in cells and an important transducer of signals. As all P-type ATPases, the Na^+^/K^+^ pump derives energy from ATP hydrolysis.

Herein, we report and compare the effects of nine different POTs (Fig. 1 and Table 1) on the *in vitro* activity of Ca^2+^-ATPase from SR. For the first time, we investigate the effects of POTs on the process of epithelial chloride secretion, energized by the activity of basolateral Na^+^/K^+^-ATPase, using an *ex vivo* model obtained from basal membrane of epithelial skin (killifish). Putative correlations between the inhibitory activity of POTs (IC_50_ values), their charge density and size were derived. The results reveal that some POTs are potent inhibitors of P-type ATPases even under almost physiological conditions (*ex vivo* study) and therefore should be taken into consideration as P-type ATPase targeting drugs. One POT, namely K_9_(C_2_H_8_N)_5_[H_10_Se_2_W_29_O_103_] (Se_2_W_29_) showed clear selectivity towards one pump (Ca^2+^-ATPase), whereas other POTs like the Anderson archetype Na_6_[TeW_6_O_24_] showed very low inhibition on both ion pumps.

**Table 1:** Structural/molecular features of the POTs used in this study

POTs (abbreviated)	Sum formula	*M*_r_	Charge	POT archetype	Ref.
P_2_W_18_	K_6_[α-P_2_W_18_O_62_]·14H_2_O	4849.83	6–	Dawson	35
TeW_6_	Na_6_[TeW_6_O_24_]·22H_2_O	2148.56	6–	Anderson-Evans	36
CoW_11_Ti	K_6_H_2_[TiW_11_CoO_40_]·13H_2_O	3239.62	8–	Mono-substituted Keggin	37
AsW_9_	Na_9_[B-α-AsW_9_O_33_]·27H_2_O	2950.37	9–	Tri-lacunary Keggin	38
SiW_9_	Na_10_[A-α-SiW_9_O_34_]·16H_2_O	2744.52	10–	Tri-lacunary Keggin	35
P_2_W_12_	K_12_[α-H_2_P_2_W_12_O_48_]·16H_2_O	3795.19	12–	Lacunary Dawson	35
As_2_W_19_	K_14_[As_2_W_19_O_67_(H_2_O)]·23H_2_O	5694.15	14–	Doubled anion based on tri-lacunary Keggin anions	39
Se_2_W_29_	K_9_(C_2_H_8_N)_5_[H_10_Se_2_W_29_O_103_]·30H_2_O	8270.09	14–	Lacunary anion based on two tri-lacunary Keggin anions containing {(WO_7_)W_4_} pentagonal unit	40
W_22_	Na_12_[H_4_W_22_O_74_]·50H_2_O	6409.10	12–	Dimeric isopolyanion based on two {W_11_} units	41

## Experimental section

### Polyoxometalates

The POTs used in this study, K_6_[α-P_2_W_18_O_62_]·14H_2_O,^35^ Na_6_[TeW_6_O_24_]·22H_2_O,^36^ K_6_H_2_[TiW_11_CoO_40_]·13H_2_O,^37^ Na_10_[α-SiW_9_O_34_]·16H_2_O,^35^ Na_9_[α-AsW_9_O_33_]·27H_2_O,^38^ K_12_[α-H_2_P_2_W_12_O_48_]·16H_2_O,^35^ K_14_[As_2_W_19_O_67_(H_2_O)]·23H_2_O,^39^ K_9_(C_2_H_8_N)_5_[H_10_Se_2_W_29_O_103_]·30H_2_O^40^ and Na_12_[H_4_W_22_O_74_]·50H_2_O^41^ (Table 1 and Fig. 1), were synthesized according to published procedures (see references in Table 1) and their identity was confirmed by infrared spectroscopy. Stock solutions of POTs were freshly prepared by dissolving the solid compound in water and keeping the solution on ice to avoid POT decomposition. The concentrations of the stock solutions were 10 mM and 1 mM for all POTs except for Se_2_W_29_ (1 mM and 0.1 mM).

### Preparation of sarcoplasmic reticulum Ca^2+^-ATPase vesicles

All reagents used for the preparation of the calcium pump vesicles were purchased from Sigma-Aldrich (Portugal). Isolated sarcoplasmic reticulum vesicles (SRVs), prepared from rabbit skeletal muscles as described elsewhere,^33^ were suspended in 0.1 M KCl, 10 mM HEPES (pH 7.0), diluted 1 : 1 with 2.0 M sucrose and frozen in liquid nitrogen for storage at –80 °C. The protein concentration was determined spectrophotometrically at 595 nm in the presence of 0.125% of sodium dodecyl sulphate (SDS) by Bradford method with bovine serum albumin as a standard. The percentage of each protein present in the SRV preparations was determined by densitometry analysis of SDS-PAGE (7.5% acrylamide) protein bands. The SR Ca^2+^-ATPase constituted at least 70% of the total protein amount in the SR-vesicles according to SDS-PAGE. The sarcoplasmic reticulum Ca^2+^-ATPase-1 (SERCA-1) was the predominant isoform in our SR preparations.^42^

### Effects of POTs on ATP hydrolysis of SR Ca^2+^-ATPase

**Scheme 1:**
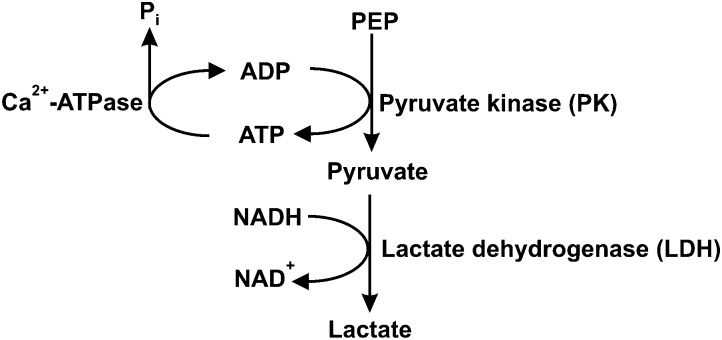
Coupled enzymatic assay for Ca^2+^-ATPase activity. PEP – phosphoenolpyruvate; P_i_ – inorganic phosphate.

Steady-state assays of the SR Ca^2+^-ATPase were measured spectrophotometrically at 25 °C using the coupled enzyme pyruvate kinase/lactate dehydrogenase assay (Scheme 1) as described elsewhere.^42^ Briefly, after the addition of the enzymes (pyruvate kinase and lactate dehydrogenase) and the substrate phosphoenolpyruvate to the medium, the experiment was initiated by adding NADH (0.25 mM) and the vesicles containing Ca^2+^-ATPase (10 μg mL^–1^).

ATP (2.5 mM) was added and the absorbance was recorded for about 1 minute (basal activity). Afterwards, the calcium ionophore calcimycin 4% (w/w), which releases again the Ca^2+^ ions, that were pumped in by the ATPase, was added and the decreasing NADH absorbance at 340 nm was measured for about 2 minutes (uncoupled ATPase activity). This was done to increase the ATPase activity (due to the ionophore-mediated impairment of the Ca^2+^ gradient) in order to better study the effect of the inhibitors and to ensure that the SR Ca^2+^-ATPase vesicles are not leaky. For the experiments including POTs, freshly prepared POT solutions (10 mM and 1 mM for all POTs except for Se_2_W_29_ 1 mM and 0.1 mM) were added to the medium prior to the addition of SR Ca^2+^-ATPase. The ATPase activity and its inhibition was measured taking into account the decrease of the OD (optical density) per minute in the absence (100%) and in the presence of the investigated POTs.^33^ The detection system was not affected by the POTs themselves (not even at their highest concentrations), which was confirmed by a rapid decrease in absorbance at 340 nm upon addition of 40 μM ADP after the assay. All experiments were performed at least in triplicates. The inhibitory power of the investigated POTs was evaluated by determining the respective IC_50_ value, that is, the POT concentration needed to induce a 50% inhibition of the Ca^2+^-ATPase enzyme activity.

### Animals used for *ex vivo* studies

Killifish (*F. heteroclitus*, 4–8 g) were collected with fish traps from the saltmarshes of Ria Formosa (Faro, Portugal) and maintained in Ramalhete Marine Station (CCMar, University of Algarve, Faro, Portugal) with running seawater (35 ppt) at a density of <5 kg m^–3^, 18–20 °C and 12 : 12 h light : dark photoperiod. The animals were handfed twice daily (final ratio of 2% of the body weight) with commercially available dry pellets (Sorgal, Portugal). The fishes were then food deprived for 24 h before sampling. The animal collections (ICN, Portugal) and the experimental procedures comply with the guidelines of the European Union Council (86/609/EU) for the use of laboratory animals. All animal protocols were performed under a “Group C” license from the Direcção-Geral de Veterinária, Ministério da Agricultura, do Desenvolvimento Rural e das Pescas, Portugal.

### Epithelial short circuit current in Ussing chambers *ex vivo*

Epithelial tissues can transport ions and generate a transepithelial voltage termed “active transport potential”, which is caused by the asymmetric distribution of ion channels and transporters on the apical and basolateral membranes. The net movement of charges from the apical to the basolateral side (and *vice versa*) generates a voltage equal to the voltage differences between the apical and basolateral membranes. *Ex vivo*, the short circuit current (*I*_sc_) is an accurate reflection of the secretory/absorptive capacity of the tissue when short-circuited. In the opercular epithelium of killifish used for our studies, *I*_sc_ is a direct measure of apical chloride secretion mediated by chloride channels, which relies on an intact basolateral Na^+^/K^+^-ATPase function.^43^,^44^

Methodology for the *ex vivo* opercular epithelia preparation followed our current methods.^45^ Fish were anaesthetized with 2-phenoxyethanol (1 : 2000 v/v), sacrificed by decapitation and the cranium was cut longitudinally. The gills and other tissue remains were removed carefully and the epithelial skin covering the opercular bone were dissected out and transferred to fresh-gassed saline (99.7 : 0.3 O_2_/CO_2_) with the following composition (all values in mM): NaCl, 160; MgSO_4_, 0.93; NaH_2_PO_4_, 3.0; CaCl_2_, 1.5; NaHCO_3_, 17.85; KCl, 3.0; glucose, 5.5; HEPES (pH 7.8), 5.0. The epithelia were overlaid onto a thin bore polythene net, protected between two parafilm gaskets and pinned over the circular aperture of a tissue holder (P2410, 0.20 cm^2^, Physiological Instruments, San Diego, USA), with the perimeter area lightly greased with vacuum silicone to minimize tissue edge damage. The mounted tissue was positioned between the two halves of the Ussing chamber (P2400, Physiological Instruments, San Diego, USA) with 4 mL of gassed saline at 22 °C and gassed with a 99.7 : 0.3 O_2_/CO_2_ mix to provide oxygenation, good mixing by gas lift and pH control (pH = 7.8).

**Fig. 2:**
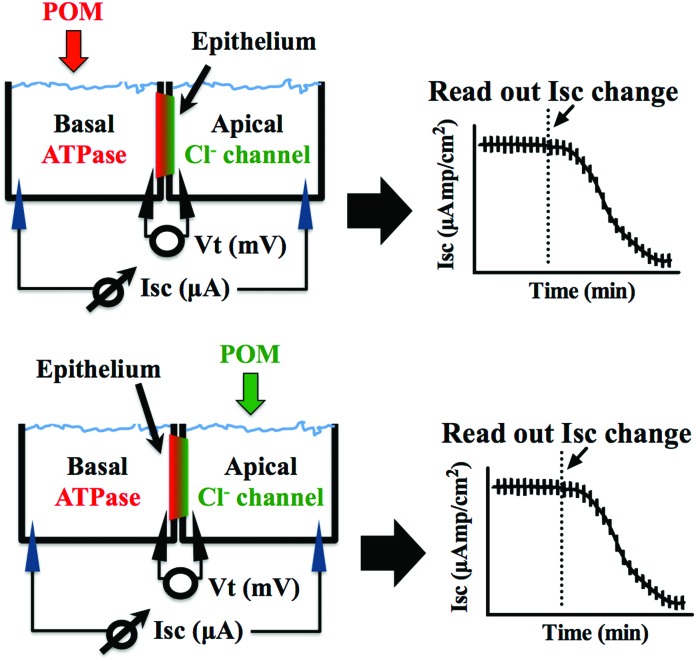
Graphic representation of the experimental setup of the opercular epithelium of killifish used for *ex vivo* studies. *I*_sc_ was measured in voltage clamp, and in this model represents chloride secretion. The process is energized by basolateral Na^+^/K^+^-ATPase and chloride is secreted apically *via* a chloride channel. In this polarised epithelium, both mechanisms are required to be intact to sustain the process of secretion.

The preparations were left to stand for at least 60 min or until a steady basal measurement of bioelectrical variables was achieved. Measurement of the short circuit current (*I*_sc_, μA cm^–2^) was performed at symmetric conditions under voltage clamp to 0 mV. The open circuit potential (*V*_t_, mV) and *I*_sc_ were monitored by means of Ag/AgCl electrodes connected to the chambers by 3 mm bore agar bridges (1 M KCl in 3% agar). Clamping of epithelia to 0 mV and recording of *I*_sc_ was performed by VCC600 voltage clamp amplifiers (Physiologic Instruments, San Diego, USA). Epithelial resistance (*R*_t_, Ω cm^2^) was manually calculated (Ohm's law) using the current deflections induced by bilateral 1 mV pulses of 3 s every minute. Bioelectrical data were continuously digitized through a Lab-Trax-4 (WPI, Sarasota, US) onto a Macbook laptop using Labscribe3 Software (Iworks systems, Dover, US). Upon signal baseline stabilization, freshly prepared POT solutions were added to the basal or the apical side of the chamber (Fig. 2) and the effects on the Na^+^/K^+^-ATPase from basal membrane or the chloride channel forming the apical membrane were followed for 60 to 90 minutes. The maximum inhibitory effect (in %) of the POTs on the ATPase activity and the effective time (ET_50_), which is the time necessary to reach 50% of the maximum effects (in minutes), was determined by measuring the % decrease of short circuit current (*I*_sc_, μA cm^–2^) in the absence (100% activity) and presence of the POTs. All the experiments were performed at least in triplicates. Calculations of the ET_50_ and maximum effect values were performed using GraphPad Prism version 6.00 for Macintosh (GraphPad Software, La Jolla California USA).

## Results and discussion

### Inhibition of Ca^2+^-ATPase by POTs: *in vitro* study

**Fig. 3:**
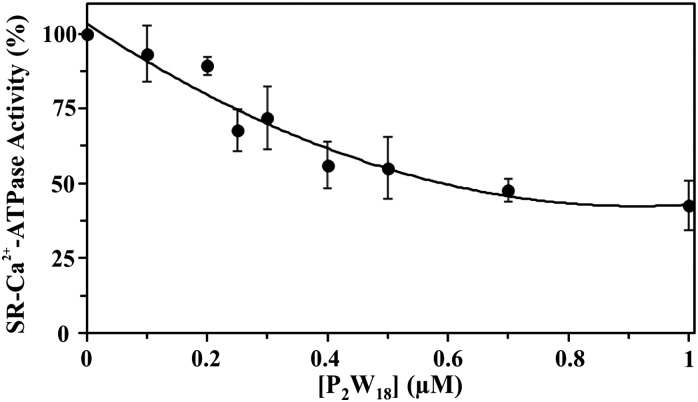
Inhibition of Ca^2+^-ATPase activity by P_2_W_18_. Ca^2+^-ATPase was determined spectrophotometrically at 340 nm and 25 °C, using the coupled enzyme pyruvate kinase/lactate dehydrogenase assay. The experiments were initiated by the addition of 10 μg mL^–1^ Ca^2+^-ATPase, in the presence or absence of 4% (w/w) of the calcium ionophore calcimycin. Data are plotted as means ± SD and fit to the equation *y* = 73.126*x*^2^ – 133.62*x* + 103.45 (*R*^2^ = 0.9424).

The effect of nine different POTs (Fig. 1 and Table 1) on the activity of SR Ca^2+^-ATPase from skeletal muscle was investigated for the first time. All of the investigated POTs inhibited Ca^2+^-ATPase activity in a concentration dependent manner. The inhibitory power of the investigated POTs was finally evaluated using IC_50_ values (Table 2). As shown in Table 2, different IC_50_ values in the μM range were determined for the various POT archetypes exhibiting different negative charges (Fig. 1 and Table 1). IC_50_ values of <1 μM were determined for the Dawson anion P_2_W_18_ (0.6 μM, Fig. 3) and the larger POT Se_2_W_29_ (IC_50_ = 0.3 μM), whereas the lowest inhibition values were observed for the isopolyanion W_22_ (IC_50_ = 68 μM) and the Anderson type TeW_6_ (IC_50_ = 200 μM). The remaining POTs exhibited IC_50_ values in the range of 1 up to 28 μM (Table 2).

**Table 2:** Inhibitory parameters of the POTs for SR Ca^2+^-ATPase (IC_50_ values) activity and Na^+^/K^+^-ATPase activity from basal membrane epithelia (effective time 50 ET_50_ and maximum inhibitory effects are calculated as the % of basal values). Inhibitory effect of different POTs applied basolaterally to the *ex vivo* preparation of the opercular epithelia of killifish mounted in Ussing chambers. Results are representative values for 3 independent experiments for each concentration (nd: not determined as no effect was observed after 30 minutes upon POT addition). Data showing the effect of ouabain for the Na^+^/K^+^-ATPase inhibition is included as a positive control for the *ex vivo* system

POTs	Ca^2+^-ATPase	Na^+^/K^+^-ATPase	
Compound name	IC_50_, (μM)	ET_50_, (min (depending on concentration of compound))	Maximum inhibition, (%)
P_2_W_18_	0.6	8.2 (0.5 μM)	86
		6.5 (1 μM)	99
		4.3 (10 μM)	100
TeW_6_	200	60 (10 μM)	10
CoW_11_Ti	4	10 (10 μM)	75
SiW_9_	16	nd	nd
P_2_W_12_	11	nd	nd
As_2_W_19_	28	nd	nd
Se_2_W_29_	0.3	6.5 (1 μM)	14
W_22_	68	nd	nd
AsW_9_	20	8.5 (10 μM)	66
Ouabain	—	3.2 (10 μM)	100

**Fig. 4:**
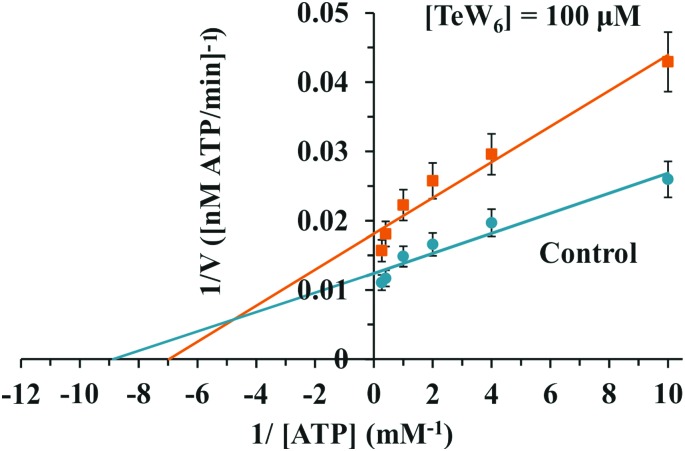
Lineweaver–Burk plots of Ca^2+^-ATPase activity in the absence (blue) and in the presence (orange) of 100 μM TeW_6_. The Anderson type TeW_6_ presented a mixed type of enzyme inhibition. Data are plotted as means ± SD. The equations for linear fits are *y* = 0.0014*x* + 0.0124 (*R*^2^ = 0.9333) for control and *y* = 0.0026*x* + 0.0181 (*R*^2^ = 0.955) for TeW_6_. The results shown are the average of triplicate experiments.

Similar moderate IC_50_ values for SR Ca^2+^-ATPase activity were previously reported for two isostructural polyanions, decaniobate [Nb_10_O_28_]^6–^ (IC_50_ = 35 μM) and decavanadate [V_10_O_28_]^6–^ (IC_50_ = 15 μM).^33^,^46^ Both decaniobate and decavanadate showed a non-competitive inhibition for Ca^2+^-ATPase activity regarding the natural ligand MgATP.^33^ Thus, to further investigate the kind of interactions between the POTs and ATPase, the type of inhibition was determined for P_2_W_18_ and TeW_6_. It was observed that both P_2_W_18_ (data not shown) and TeW_6_ exhibited a mixed type inhibition suggesting that they can interact with the substrate-bound form of Ca^2+^-ATPase (Fig. 4). The binding site of V_10_ on Ca^2+^-ATPase was previously described, which involves at least three protein domains, including the phosphorylation and the nucleotide binding sites.^47^

### Inhibition of Na^+^/K^+^-ATPase by POTs: *ex vivo* study

POTs were added to the basolateral side of epithelia (Fig. 2) to characterize their effects on the Na^+^/K^+^-ATPase activity. The obtained results, which are summarized in Table 2, revealed inhibition rates ranging from only 10% for TeW_6_ (at 10 μM) to almost 100% for P_2_W_18_ (at 1 and 10 μM).

**Fig. 5:**
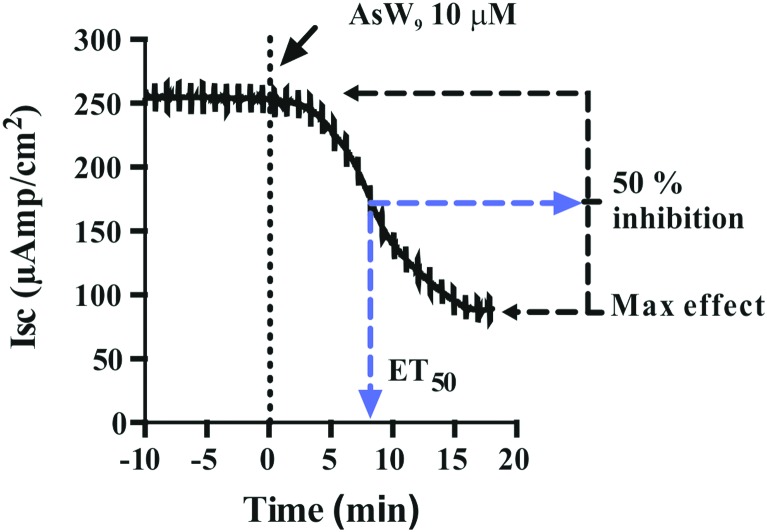
The effect of the Keggin type AsW_9_ applied in basolateral membranes at a concentration of 10 μM is shown. Original trace of the effect of short circuit current (*I*_sc_, μA cm^–2^) in the opercular epithelium of killifish mounted in Ussing chambers and kept under voltage clamp (*V*_t_ = 0 mV). Effective time 50 (ET_50_) and maximum inhibitory effects are calculated as the % of basal values. Both parameters were calculated for three individual independent experiments and used to generate Table 2. An arrow indicates the time of POT application and consequently the time point zero. Time with negative values represents stable basal control periods.

It should be noted that the heteropoly POTs SiW_9_, P_2_W_12_, As_2_W_19_ as well as the isopoly POT W_22_ showed no effect on ATPase activity after 30 minutes upon addition of 10 μM compound. In order to further characterize the inhibitory effects of the investigated POTs on Na^+^/K^+^-ATPase, we calculated the maximum inhibitory effects with respect to the basal values. In addition, we calculated the effective time (ET_50_), defined as the time necessary to achieve 50% of the maximum effect, to have a measure for the inhibitory dynamics of the individual POTs (Fig. 5). In Fig. 5 a constant height of the current deflections used to calculate the tissue resistance can be observed, indicating that the *ex vivo* preparations retained their integrity and selectivity before and after POT exposure. Therefore, since no changes were observed on the tissue resistance (*i.e.* tissue integrity) all observed effects of POT on the epithelial function are due to changes of the short circuit current (*I*_sc_). Modification of *I*_sc_ provides an immediate read-out of inhibitory/stimulatory effects on either the apical chloride channel or the basolateral Na^+^/K^+^-ATPase (Fig. 2). For example, AsW_9_ (at 10 μM) exhibits a maximum inhibition of 66% of the basal current and an ET_50_ value of 8.5 min (Fig. 5). It has to be noted that both the maximum inhibitory effect (providing information about inhibitor efficacy) and ET_50_ (providing information about inhibition velocity) are necessary to define the biological effects of POTs (Fig. 5 and Table 2). For the *ex vivo* studies, a positive control experiment was performed with the conventional Na^+^/K^+^-ATPase inhibitor ouabain.^43^,^49^ Ouabain (at 10 μM) showed a maximum inhibition value of 100% and an ET_50_ of 3.2 minutes (Fig. S1, ESI†). By inhibiting the basolateral Na^+^/K^+^-ATPase activity, ouabain concomitantly prevents apical chloride secretion in the studied epithelia model as this process is energized by Na^+^/K^+^-ATPase.^43^,^49^

**Fig. 6:**
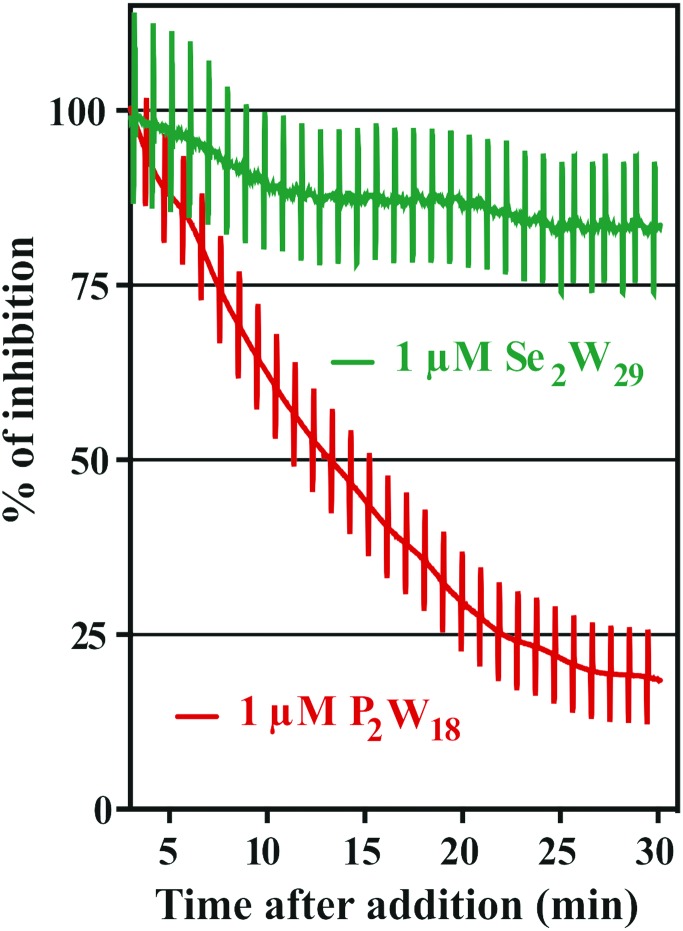
Inhibition (%) of the Na^+^/K^+^-ATPase from basal membrane of the skin epithelia by two POTs P_2_W_18_ and Se_2_W_29_. The inhibition rate of P_2_W_18_ (1 μM) was 82% after 30 minutes (red), whereas for the most potent Ca^2+^-ATPase inhibitor so far described, Se_2_W_29_ (IC_50_ = 0.3 μM), a minor effect (green) was observed (14% inhibition, after 30 minutes). Perpendicular lines were used to calculate tissue resistance. As it can be observed, the *ex vivo* epithelia preparations retained integrity and selectivity after POT exposure.

The addition of POTs to apical saline had no effect on *I*_sc_ and therefore ruling out chloride channels as putative POT targets, at least at POT concentrations up to 10 μM. The largest POT (in terms of volume and number of addenda atoms) under investigation, Se_2_W_29_, exhibited the highest inhibition (IC_50_ = 0.3 μM) of SR Ca^2+^-ATPase activity during the *in vitro* study. However, used at the same concentration (1 μM), it was one of the weakest inhibitors (14% inhibition; Table 2 and Fig. 6) for the Na^+^/K^+^-ATPase activity during the *ex vivo* study. In contrast, P_2_W_18_ efficiently inhibited both the SR Ca^2+^-ATPase *in vitro* (IC_50_ = 0.6 μM) and the Na^+^/K^+^-ATPase *ex vivo* (99% inhibition) (Table 2 and Fig. 6). In fact, P_2_W_18_ was demonstrated to be as potent as ouabain in inhibiting the Na^+^/K^+^-ATPase activity (Table 2). The remaining studied POTs showed similar inhibitory effects *in vitro* and *ex vivo*. For example, the potential of TeW_6_ to inhibit Ca^2+^-ATPase (IC_50_ value of 200 μM) was as low as its effect against Na^+^/K^+^-ATPase (inhibition of 10%; Table 2).

Both experiments (*in vitro* and *ex vivo*) clearly demonstrated the high selectivity of Se_2_W_29_ for inhibiting the Ca^2+^ pump due to its rather sobering *ex vivo* results rendering this POT not the best choice to target the Na^+^/K^+^-pump *in vivo*. The size (in terms of volume and number of addenda atoms) of this large POT could be one aspect affecting the kinetics of its cellular uptake, thus preventing the POT from targeting the enzyme. The mechanisms of POT uptake and their permeation through epithelia still need to be clarified. The same selectivity pattern for inhibition of Ca^2+^-ATPase activity (IC_50_ = 400 μM)^33^ over Na^+^/K^+^-ATPase (IC_50_ = 1.5 mM)^34^ was shown in previous studies for orthotungstate (HWO_4_^–^). Therefore, it seems that POT-mediated inhibition is pump-specific and there is no POT structure that is perfectly suited for all ion pumps in general. Moreover, the *ex vivo* results show that not only the affinity of the inhibitory compound is relevant, but also how the POT gains access to the inhibition site within an intracellular compartment, rendering the POT-Na^+^/K^+^-ATPase interaction a complex one. The presented combination of *in vitro* and *ex vivo* studies using two different models to study the effects of POTs on the activity of ATPases indicates the importance of establishing experimental conditions to be as close to the physiological environment as possible.

The majority of P-type ATPase inhibitors in therapy target the Na^+^/K^+^-ATPase.^32^,^48^ These compounds, which are used for the treatment of several diseases such as heart failure, psychosis, malaria and bacterial infection, show inhibitory capacities resembling those of the here investigated POTs.^48^ Only a few kinetic studies have been described so far testing POTs as P-type ATPase inhibitors.^24^,^32^,^42^,^46^ The *in vitro* inhibition of Na^+^/K^+^-ATPase by the Keggin POTs H_3_PW_12_O_40_ and H_4_SiW_12_O_40_ was previously described reporting IC_50_ values between 3 to 4 μM^32^ although information about the type of inhibition and the mechanism of action are still lacking. In this study the comparable IC_50_ value for the isostructural Keggin POT CoW_11_Ti (IC_50_ = 4 μM) was observed.

### Protein interactions and structure/function features of POTs

**Fig. 7:**
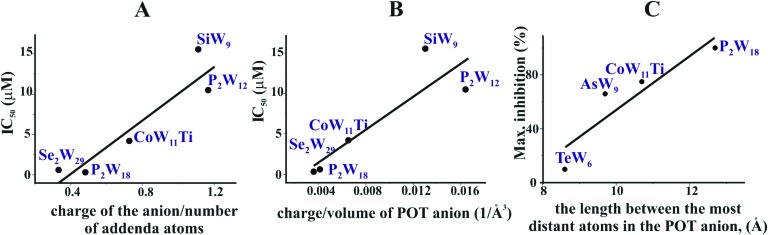
Structure–activity correlations of different POTs for ATPases inhibition. (A) Correlation between the IC_50_ values of five POTs (IC_50_ lower then 16 μM) of Ca^2+^-ATPase inhibition and their charge density expressed as charge of the POT divided by its number of W atoms. (B) Correlation between the IC_50_ values of five POTs (IC_50_ lower then 16 μM) against Ca^2+^-ATPase and the charge density expressed as charge of the POT divided by its volume (in Å^–3^). (C) Correlation between the percentage of maximum inhibition of four POTs (applied with the same concentration of 10 μM) against Na^+^/K^+^-ATPase and POT size expressed as the length between the most distant atoms in the POT anion (in Å).

In order to decipher specific features of the nine POTs that are responsible for the inhibition of Ca^2+^-ATPase, we correlated the POT parameters like size and charge density with their IC_50_ values of inhibition (Fig. 7A and B). No correlation was found when considering all nine POTs and thus all determined IC_50_ values. However, when taking only into account the high affinity POTs, exhibiting IC_50_ values lower then 16 μM, we observed a correlation between their activity (IC_50_ value) and their charge density, which was defined as charge of the POT divided by its number of W atoms (Fig. 7A) as well as by the volume of POT anion (Fig. 7B). As can be deduced from this data, POTs such as Se_2_W_29_ and P_2_W_18_ with a low charge density (Fig. 7A and B) favored the inhibition of Ca^2+^-ATPase activity indicating that besides electrostatic interactions also steric interactions (depending on the shape complementarity between the POT and the inhibition site) might play important roles in the successful inhibition of Ca^2+^-ATPase.

For the *ex vivo* results (Na^+^/K^+^-ATPase) no correlation between the ET_50_ values and POT charge density was observed. However, a dependency of the maximum inhibition of four POTs on their size, defined as length between the most distant atoms in the POT anion was found (Fig. 7C).

**Fig. 8:**
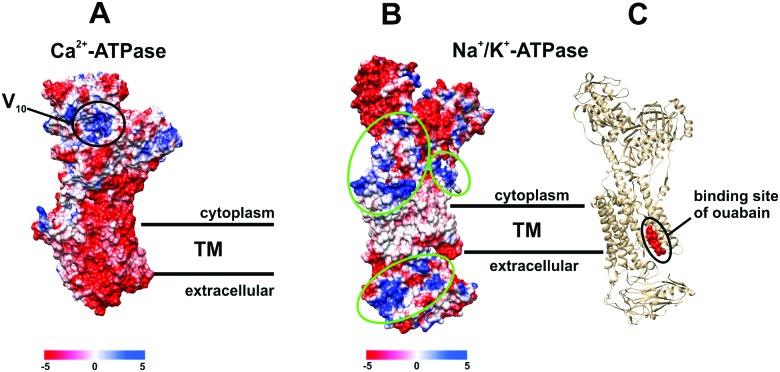
Electrostatic (Coulomb) potential surface presentation (scale in kcal mol^–1^ e^–1^, TM – transmembrane area) of two P-type ATPases: (A) Ca^2+^-pump in E2 conformation^31^,^32^ (PDB entry: ; 1KJU) showing the proposed binding site of decavanadate V_10_;^47^ (B) electronic (coulombic) surface representation of the Na^+^/K^+^-pump (green circles indicate potential binding sites for POMs); (C) structure of the Na^+^/K^+^-ATPase-ouabain complex^31^ (PDB entry: ; 3A3Y). Na^+^/K^+^-ATPase is illustrated as beige cartoon, whereas bound ouabain is depicted in sphere mode.

POTs are also known to be strong kinase and phosphatase inhibitors by acting through noncovalent interactions, which is indispensable for the usage of POMs in the therapy of various diseases.^17^–^19^,^26^,^27^ It was demonstrated that decavanadate V_10_ exhibits specific interactions with SR Ca^2+^-ATPase, which is supposed to be non-competitive with respect to ATP and induces protein cysteine oxidation with concomitant vanadium reduction explaining the high inhibitory capacity of V_10_ (IC_50_ = 15 μM).^24^,^33^,^47^,^48^ The V_10_ binding site, which is formed by three protein domains,^47^ is located at the cell cytoplasmatic side (Fig. 8A). V_10_ can interact with proteins by electrostatic interactions or by hydrogen bonding and the specific residues involved in V_10_-SR Ca^2+^-ATPase interaction still need to be established, but might include the oxidized cysteine.^24^ In contrast to monomeric vanadate, which only binds to the E2 conformation, V_10_ binds to all protein conformations, E1, E1P, E2 and E2P,^33^ indicating the possibility of V_10_-ATPase interactions at the extracellular side of the enzyme.^48^ The region where V_10_ is expected to bind exhibits a positively charged surface and could therefore also be addressed by other negatively charged POMs (Fig. 8A).

Many drugs are known to act as ionic pumps inhibitors, such as ouabain, omeprazole or thapsigargin, but only for some of these compounds like ouabain the mechanisms and protein binding sites were clearly established.^32^ According to structural analysis, ouabain inhibits the Na^+^/K^+^-ATPase through binding to a cavity formed by transmembrane helices (Fig. 8C, PDB entry: ; 3A3Y).^32^ The binding sites for POMs are not known yet, however, considering the structures of POMs and ouabain, it is very unlikely that they share the same binding site (within the neutral transmembrane area). Analysis of the electrostatic (coulombic) surface of Na^+^/K^+^-ATPase reveals that both the cytoplasmic and extracellular region of the enzyme possess areas exhibiting a positive surface potential (Fig. 8B),^31^ which could be addressed by the negatively charged POMs, the binding sites of which need to be identified.

## Conclusions

In general, polyoxometalates are able to inhibit phosphatases, ecto-nucleotidases and P-type ATPases. Here, we demonstrated that the Ca^2+^-ATPase activity from sarcoplasmic reticulum is inhibited by several POTs. P_2_W_18_ was the most potent ATPase inhibitor in this study as it exhibited the highest inhibitory activity for the Na^+^/K^+^-ATPase (100% inhibition at 10 μM) and the second highest for the Ca^2+^-ATPase (IC_50_ = 0.6 μM). A mixed type of inhibition was observed for P_2_W_18_ and TeW_6_ suggesting a different mode of protein interaction with Ca^2+^-ATPase activity than those observed for decavanadate and decaniobate (non-competitive inhibitors). The most potent Ca^2+^-ATPase inhibitor Se_2_W_29_ showed only limited effects on the Na^+^/K^+^-ATPase from basal membrane of the skin epithelia demonstrating that some POTs exhibit selectivity against certain ion pumps. The here reported *ex vivo* model of Na^+^/K^+^-ATPase was used for the first time to study the effects of POTs on the processes of epithelial chloride secretion, energized by the activity of the basolateral Na^+^/K^+^-ATPase. Finally, we were able to derive structure–activity relationships for high affinity POTs (IC_50_ < 16 μM) indicating that the inhibition potential of the POTs is correlated with their charge density, which will help to clarify their different inhibitory activity. Polyoxotungstates are promising inorganic inhibitors of P-type ATPases although their potential *in vivo* applications require more studies and toxicological information.

## Conflicts of interest

There are no conflicts to declare.

## Supplementary Material

Supplementary information
