# Supplementary material for: The P-type ATPase inhibiting potential of polyoxotungstates
Source: Metallomics. 2018 Jan 5;10(2):287–95. doi: 10.1039/c7mt00279c (PMC5824666; doi:10.1039/c7mt00279c)
Supplement: Supplementary file 1 [file MT-010-C7MT00279C-s001.pdf]

Electronic Supplementary Material (ESI) for Metallomics.
This journal is © The Royal Society of Chemistry 2017

ELECTRONIC SUPPLEMENTARY INFORMATION

The P-type ATPase inhibiting potential of polyoxotungstates

Nadiia Gumerova[a], Lukáš Krivosudský[a,b], Gil Fraqueza[c,d], Joscha Breibeck[a], Emir Al-Sayed[a], Elias Tanuhadi[a], Aleksandar Bijelic[a], Juan Fuentes[c], Manuel Aureliano[c,e]* and Annette Rompel[a]*

[a] *Universität Wien, Fakultät für Chemie, Institut für Biophysikalische Chemie, Althanstr. 14, 1090 Wien Austria, http://www.bpc.univie.ac.at; E-mail: annette.rompel@univie.ac.at.*

[b] *on leave from: Comenius University, Faculty of Natural Sciences, Department of Inorganic Chemistry, Mlynská dolina, Ilkovičova 6, 842 15 Bratislava, Slovakia.*

[c] *Centre of Marine Sciences, University of Algarve, 8005-139 Faro, Portugal.*

[d] *Institute of Engineering, University of Algarve, 8005-139 Faro, Portugal.*

[e] *Faculty of Sciences and Technology, University of Algarve, 8005-139 Faro, Portugal, E-mail: maalves@ualg.pt.*

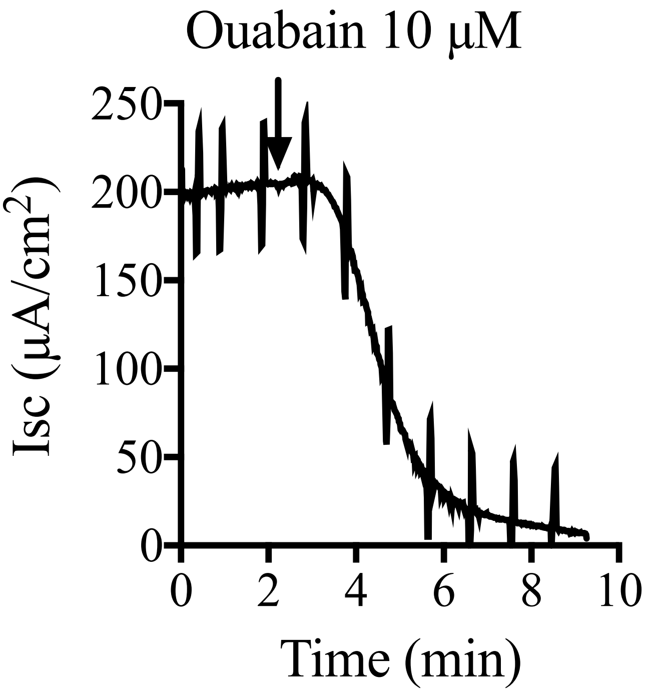

Figure S1. Original trace for ouabain of the effect of short circuit current (Isc, µA/cm^2) in the opercular epithelium of killifish mounted in Ussing chambers and kept under voltage clamp (Vt = 0 mV). Effective time 50 (ET_{50}) and maximum inhibitory effects were calculated based on three independent experiments. Ouabain (10 µM) showed a maximum inhibition value of 100 % and an ET_{50} of 3.2 ± 0.2 minutes.
